# Regulation of Apetala2/Ethylene Response Factors in Plants

**DOI:** 10.3389/fpls.2017.00150

**Published:** 2017-02-21

**Authors:** Ujjal J. Phukan, Gajendra S. Jeena, Vineeta Tripathi, Rakesh K. Shukla

**Affiliations:** ^1^Biotechnology Division, CSIR-Central Institute of Medicinal and Aromatic PlantsLucknow, India; ^2^Botany Division, CSIR-Central Drug Research InstituteLucknow, India

**Keywords:** AP2/ERF, ubiquitination, transcriptional regulation, translational regulation, retrograde regulation

## Abstract

Multiple environmental stresses affect growth and development of plants. Plants try to adapt under these unfavorable condition through various evolutionary mechanisms like physiological and biochemical alterations connecting various network of regulatory processes. Transcription factors (TFs) like APETALA2/ETHYLENE RESPONSE FACTORS (AP2/ERFs) are an integral component of these signaling cascades because they regulate expression of a wide variety of down stream target genes related to stress response and development through different mechanism. This downstream regulation of transcript does not always positively or beneficially affect the plant but also they display some developmental defects like senescence and reduced growth under normal condition or sensitivity to stress condition. Therefore, tight auto/cross regulation of these TFs at transcriptional, translational and domain level is crucial to understand. The present manuscript discuss the multiple regulation and advantage of plasticity and specificity of these family of TFs to a wide or single downstream target(s) respectively. We have also discussed the concern which comes with the unwanted associated traits, which could only be averted by further study and exploration of these AP2/ERFs.

## Introduction

Plants are mostly affected by multiple stresses like salinity, toxicity, pathogen attack, and wounding. To survive and reproduce in adverse conditions, plants develop various adaptive traits governed through a complicated system. The interconnected and diverse networks operated during downstream signaling cascades are regulated by TFs which bind to the cis elements present in their promoters (Papdi et al., 2015). AP2/ERFs are one of the most important families of TF in plants which regulate various developmental and stress responsive pathways (Licausi et al., 2013; Li et al., 2015). The specific and/or differential binding affinity of these AP2/ERFs to multiple cis-elements and other proteins enable them to respond to different regulatory processes simultaneously (Shoji et al., 2013; Wang L. et al., 2014). In this review we will highlight the multiple roles of AP2/ERFs in regulating diverse plant processes, their mechanism of action, auto/cross-regulation properties and differential interaction at protein and DNA level. We will also discuss how AP2/ERF family of TFs are regulated by different plant growth regulators (PGRs) and their role in retrograde signaling. With multiple responses there comes both positive and negative regulation which needs proper concern before generation of recombinant plants. These unexplored detrimental effects need to be addressed first for commercialization of such plants. Our objective is to focus on the recent advancements made in this area to the best of our knowledge in order to make a comprehensive review on the roles of this family of proteins in plants.

## AP2/ERF transcription factors

AP2/ERFs are mainly plant-specific TFs with 122 and 139 ERF family genes in *Arabidopsis* and rice respectively, although it has been speculated that they might have been descended from non-plant organisms (Nakano et al., 2006). Number of AP2/ERFs present in different plants are: *Triticum* spp.- 117 (Zhuang et al., 2011), *Populus* spp.- 200 (Zhuang et al., 2008), *Brassica* spp.- 291 (Song et al., 2013, 2016), *Vitis* spp.- 149 (Ito et al., 2014), *Citrus* spp.- 108 (Licausi et al., 2010), *Solanum* spp.- 155 (Charfeddine et al., 2015), *Phyllostachys* spp.- 116 (Wu et al., 2015), *Daucus* spp.- 267 (Li et al., 2015), *Eucalyptus* spp.- 209 (Cao et al., 2015), *Salix* spp.- 173 (Rao et al., 2015), *Cassava* spp.- 147 (Fan et al., 2016), *Barley* spp.- 121 (Guo et al., 2016) *Gossypium* spp.- 271 (Lei et al., 2016) and *Bamboo* spp.- 142 (Huang Z. et al., 2016). His and Asn rich HNH class of homing endonucleases from *Tetrahymena thermophile* (Eukaryote ciliate), *Trichodesmium erythraeum* (cyanobacterium), Enterobacteria phage *RB49* (virus), and Bacteriophage *Felix 01* (Virus) are reported to have an *AP2* domain (Magnani et al., 2004; Wuitschick et al., 2004). Homing endonucleases promote high-frequency lateral transfer of the intervening sequence, which might explain the origin and gene exchange theory between non-plant organisms and plants (Wessler, 2005). Most of the AP2/ERFs lack introns which further supports the hypothesis that they are supposed to be evolved from a prokaryote after horizontal gene transfer (Magnani et al., 2004). Based on DNA binding domain (DBD), AP2/ERFs are classified into AP2, RAV (related to *Abscisic acid insensitive3/Viviparous1*), DREB (subgroup A1–A6), ERF (subgroup B1-B6), and others (Sakuma et al., 2002). Due to their ability to bind with the multiple cis-elements with their highly conserved DBD this member of protein family is likely to be involved in multiple responses in plants. Earlier Dehydration Responsive Element Binding Proteins (DREB) were thought to impart tolerance to cold, dehydration and salt stress through DRE/CRT elements while ERF leads to biotic stress tolerance through GCC-box element (Shinozaki and Yamaguchi-Shinozaki, 2003; Guo and Ecker, 2004; Huang et al., 2008). But now many DREBs (CBF1, TINY, HARDY) and ERFs (RAP2.4, ERF1, HRE2, ThERF1, TdSHN1) have been identified that are activated by different interconnected pathways which binds to both DRE and GCC elements as shown in Table 1 (Sun et al., 2008; Yang et al., 2009; Cheng et al., 2013; Wang L. et al., 2014; Zhu et al., 2014; Djemal and Khoudi, 2015; Lee et al., 2015). One of the probable reasons for multiple regulatory responses are due to their differential binding to the promoters, they can mediate simultaneous regulation of multiple responses. But the mechanism of this complicated regulation is still far from being fully elucidated. The mechanism identified in a plant may vary for a homolog in another plant. Some recent reports suggest that AP2/ERFs can regulate both abiotic and biotic stress in plants (Li X. et al., 2014; Li Z. et al., 2014; Jisha et al., 2015; Tian et al., 2015; Zhang et al., 2015; Figueroa-Yanez et al., 2016; Romero et al., 2016; Wang X. et al., 2016). In one of our study an AP2/ERF (*PsAP2*) from Poppy which bind to both DRE as well as GCC box elements thus provide abiotic and biotic stress tolerance in tobacco (Mishra et al., 2015). The crosstalk in the signaling pathways or interaction/stimuli dependent activation or differential binding affinity could be the keys in regulational cascades of above mentioned ERFs (Eini et al., 2013; Pandey et al., 2015; Zeng et al., 2015).

**Table 1:** **List of AP2/ERFs that show multiple developmental and stress responses by binding to various cis-elements**.

**Plant species**	**Gene name**	**Interacting motifs**	**Regulated traits**	**References**
*Arabidopsis thaliana*	*TINY*	DRE/CRT & GCC	Negatively regulates development, positively regulates abiotic stress	Sun et al., 2008
*Arabidopsis thaliana*	*RAP2.4*	DRE/CRT & GCC	Positively regulates light- and ethylene-mediated development and drought stress response	Lin et al., 2008
*Arabidopsis thaliana*	*RAP2.4b*	DRE/CRT & GCC	Similar to RAP2.4	Akhtar et al., 2012
*Arabidopsis thaliana*	*HARDY*	DRE/CRT & GCC	Positively regulates drought and salt response	Akhtar et al., 2012
*Arabidopsis thaliana*	*CBF1*	DRE/CRT & GCC	Positively regulates Cold response	Hao et al., 2002; Yang et al., 2009
*Arabidopsis thaliana*	*ERF4*	DRE/CRT & GCC	Positively regulates ethylene and ABA responses	Yang et al., 2009
*Arabidopsis thaliana*	*RAP2.3*	DRE/CRT & GCC	Positively regulates low oxygen, oxidative, and osmotic stress responses	Yang et al., 2009
*Arabidopsis thaliana*	*ERF53*	DRE/CRT & GCC	Positively regulates heat and ABA response	Gong et al., 2008
*Arabidopsis thaliana*	*HRE2*	DRE/CRT & GCC	Positively regulates development and hypoxia response	Lee et al., 2015
*Arabidopsis thaliana*	*ERF1*	DRE/CRT & GCC	Positively regulates salt, drought, and heat response	Yang et al., 2009
*Arabidopsis thaliana*	*DREB2A*	DRE/CRT & GCC	Positively regulates drought, salt, heat, cold response	Sakuma et al., 2002
*Arabidopsis thaliana*	*ESE1*	DRE/CRT & GCC	Positively regulates alt response	Zhang et al., 2011
*Brassica napus*	*BnDREBIII-1/2/3*	DRE/CRT & GCC	Positively regulates abiotic stress response genes	Liu et al., 2006
*Triticum aestivum*	*TaERF1*	DRE/CRT & GCC	Positively regulates drought, cold, and salt	Xu et al., 2007
*Jatropha curcas*	*JcERF*	DRE/CRT & GCC	Positively regulates salt and cold response	Tang et al., 2007
*Capsicum annuum*	*CaERFLP1*	DRE/CRT & GCC	Positively regulates pathogen and salt response	Lee et al., 2004
*Glycine max*	*GmERF3*	DRE/CRT & GCC	Positively regulates pathogen, drought, and salt response	Zhang et al., 2009
*Nicotiana tabacum*	*TSI1*	DRE/CRT & GCC	Positively regulates pathogen and osmotic stress response	Park et al., 2001
*Solanum lycopersicum*	*JERF3*	DRE/CRT & GCC	Positively regulates drought, salt, cold, and osmotic stress response	Wu et al., 2008
*Nicotiana tabacum*	*NtCEF1*	DRE/CRT & GCC	Positively regulate pathogen response	Lee et al., 2004
*Tamarix hispida*	*ThERF1*	DRE/CRT & GCC	Negatively regulates abiotic stress tolerance	Wang L. et al., 2014
*Solanum lycopersicum*	*LeERF2*	DRE/CRT & GCC	Positively regulates freezing and ethylene response	Hongxing et al., 2005
*Papaver somniferum*	*PsAP2*	DRE/CRT & GCC	Positively regulates drought and salt response	Mishra et al., 2015

## Transcriptional regulation of AP2/ERFs

### Complex mode of activation

Expression of AP2/ERF and their downstream genes were tightly regulated in plants in order to maintain a perfect balance between stress and developmental responsive pathways. An example of complex transcriptional regulation was observed in *DREBs* under cold stress. Promoter of *DREB1C/CBF2* contains several cold inducible motifs such as ICEr1 (Inducer of CBF Expression region 1), ICEr2 and CM2-Conserved DNA Motif 2 (Doherty et al., 2009; Zarka et al., 2003). bHLH (Basic Helix loop Helix) ICE1 (Inducer of CBF Expression 1) binds to ICEr1 motif under cold stress and activate transcription of *DREB1C* (Chinnusamy et al., 2003). Under control condition ICE1 remains unstable because of ubiquitination by a RING (Really Interesting New Gene) finger E3 ligase (HOS1) but under cold stress polyubiquitination of ICE1 is prevented by sumoylation by SIZ1-SUMO E3 ligase (Dong et al., 2006; Miura et al., 2007). ICE1 physically interacts with a R2R3 MYB transcriptional activator MYB15 to induce expression of *DREB1/CBF* and provide cold tolerance in *Arabidopsis* (Agarwal et al., 2006). Calmodulin-binding transcription activators CAMTA3 also binds to CM2 motif present in the promoter of *DREB1/CBF* and regulate their expression in calcium-dependent manner (Doherty et al., 2009). Another motif present in *DREB1C/CBF2* promoter is G-box (CACGTG) to which a bHLH PIF7 (Phytochrome Interacting Factor) interacts and suppresses expression of *DREB1C*. PIF7 interacts with TOC1 (Timing of CAB expression 1) and PhyB (phytochrome B) to transcriptionally repress the expression (Kidokoro et al., 2009; Dong et al., 2011). *DREB1C/CBF2* are also regulated by MYB-related TFs CCA1 (Circadian Clock Associated 1) and LHY (Late Elongated Hypocotyl) that bind to the EE (AAAATATCT) and CCA1 (AATCT) motifs of *DREB1C/CBF2* promoters and positively regulate their expression (Alabadi et al., 2001; Nakamichi et al., 2009). These TFs are regulated so sophistically because under control conditions *AtDREB2A* leads to growth retardation and reduced reproduction as seen in over-expressed lines (Kim et al., 2012). Therefore, under non-stressed condition plants recruit GRF7 (Growth-Regulating Factor 7) that recognizes TGTCAGG element in the promoter of *DREB2A* and represses its expression. These complex regulation of DREBs through which they modulate different responses are mentioned in Table 2. Not only DREB2A but constitutive expression of AtDREB1A, AtERF1, OsERF1, and AtERF14 shows growth retardation which is not a desirable trait under normal condition (Onate-Sanchez et al., 2007). Another example where AP2/ERFs are tightly regulated is the regulation of *ORCA 3* (Octadecanoid Responsive *Catharanthus* AP2-Domain Protein). *ORCA3* has a regulatory role in primary and secondary metabolism in *Catharanthus roseus*, therefore it needs to be properly regulated at the transcriptional level. Its promoter carries a 74-bp long autonomous Jasmonate Responsive Element (JRE) having a quantitative sequence responsible for enhanced expression and a qualitative sequence that acts as an on/off switch in response to MeJA. CrMYC2 binds to T/G-box (CACGTG–G box with one mismatch) present in the qualitative sequence of JRE and mediate expression of *ORCA3* that in turn regulate early jasmonate-responsive genes (Zhang et al., 2011). AT-Hook DNA-binding proteins also recognize JRE element and regulate expression of *ORCA3* (Vom Endt et al., 2007). In *Arabidopsis* AtMYC2 regulate *ORCA3* by binding with JRE element and regulate JA-responsiveness (Montiel et al., 2011). There are other reports in which these ERFs are transcriptionally regulated through different responses and complex mechanisms (Cole et al., 2009; Li et al., 2011; Ogata et al., 2015). So orchestration of these TFs (DREB2A, ORCA3, and most likely other ERFs) in plants through a single cascade is not possible because of multiple interacting and regulatory partners associated with them. One way could be the use of stress-inducible promoter rather than constitutive ones, but promoter and stimuli strength would greatly influence the expression of transgene under these conditions.

**Table 2:** **Complex mode of regulation of DREBs**.

**Regulator**	**Promoter motifs**	**Regulation**	**Response**	**References**
ICE1 (Inducer of CBF Expression 1) with MYB15	ICEr1 (Inducer of CBF Expression region 1)	Activates *DREB1C/CBF2*	Cold tolerance	Chinnusamy et al., 2003
CAMTA3	CM2-Conserved DNA Motif 2	Activates *DREB1C/CBF2*	Calcium signaling	Doherty et al., 2009
PIF7 (Phytochrome Interacting Factor) with TOC1 (Timing of CAB expression 1) and PhyB (phytochrome B)	G-box (CACGTG)	Suppresses *DREB1C/CBF2*	Circadian control	Kidokoro et al., 2009; Dong et al., 2011
CCA1 (Circadian Clock Associated 1) with LHY (Late Elongated Hypocotyl)	EE (AAAATATCT) and CCA1 (AATCT) motifs	Activates *DREB1C/CBF2*	Circadian control	Alabadi et al., 2001; Nakamichi et al., 2009
GRF7 (Growth-Regulating Factor 7)	TGTCAGG motifs	Suppresses *DREB2A*	Growth control	Kim et al., 2012

### Splicing and miRNA regulation

Apart from transcriptional regulation DREBs are regulated at the post-transcriptional level. Modifications through alternative splicing have been reported in rice *OsDREB2A/2B* (Matsukura et al., 2010), barley *HvDRF1* (Xue and Loveridge, 2004), wheat *WDREB2* (Egawa et al., 2006), and maize *ZmDREB2A* (Qin et al., 2007) to regulate proper functioning of them. Under normal conditions they produce an inactive transcript containing stop codon before the DNA-binding domain while under stress they produce an active transcript encoding a full-length protein. AP2/ERFs regulate multiple processes; therefore rDNA-technology mediated transcriptional inactivation through alternate splicing may interfere with the regulation of developmental responses. So exploration of the regulatory network involved in alternate splicing is necessary to modulate post-transcriptional changes in the genetically modified plants. Another mode of regulation involves non-coding small RNA or microRNA (miRNA). It is reported that miRNA172 regulate many protein-coding AP2s such as *AT2G28550, AT5G60120*, and *AT5G67180* (Chen, 2004). They are involved in gene silencing and post-transcriptional gene regulation that occurs either via degradation of mRNA or silencing its translation. Mature miRNA often becomes a part of RNA-induced silencing complex (RISC) having Dicer and many other proteins to regulate RNA silencing. The miRNA172 regulates floral organ development and floral stem cell proliferation by acting as a potential translational repressor of AP2/ERF proteins. This regulation is very important for proper development of the reproductive organs and for the timely termination of floral stem cells. The putative miRNA172 binding sites are located within the coding regions of the genes but are outside of the conserved AP2 domains (Chen, 2004). Apart from miRNA172, other miRNAs such as miRNA156 and miRNA838 are reported to be probably involved in AP2/ERF regulation (Kavas et al., 2015). Now miRNA biogenesis is under tight temporal and spatial control, regulated at multiple levels like transcription, modification, processing in nucleus, and cytoplasm. So study of miRNA and its downstream regulation of AP2/ERF seem very important to further understand their role in different responses.

## Translational regulation of AP2/ERFs

### Phosphorylation

For proper regulation of several physiological processes in plants such as translational and post-translation modifications are key features of AP2/ERFs. Phosphorylation by kinases is one such event thereby which the AP2/ERFs are activated. Based on functional classification these kinases modify different substrates such as Serine/threonine protein kinases phosphorylate the OH group of serine or threonine and tyrosine-specific protein kinases phosphorylate tyrosine amino acid residues. Phosphorylation by different kinases usually results in a functional change of the target protein by changing activity, cellular location, or association with other proteins. For example PgDREB2A when phosphorylated at Thr residue(s) alters its binding affinity to DRE/CRT cis-element (Agarwal et al., 2007). When BWMK1 (blast and wound-induced MAP kinase1) through its TDY phosphorylation motif phosphorylates OsEREBP1 which enhances DNA-binding activity of OsEREBP1 which further activates several basic pathogenesis-related genes by binding to the GCC box element (AGCCGCC) present in their promoter (Cheong et al., 2003). Similarly JA-modulated MAPKK1 (JAM1) leads to increased transcriptional regulation of ORC1 through phosphorylation (De Boer et al., 2011). An example of kinase regulating cellular location is Histidine kinases and histidine-containing phosphotransfer proteins. Through phosphorylation it enables relocation of CRFs from the cytosol into the nucleus in response to cytokinin (Rashotte et al., 2006). Phosphorylation also affects protein-protein association as seen in the case of AtERF104. Pathogen-responsive mitogen-activated protein kinase MPK6 phosphorylates AtERF104, which is released as a substrate from MPK6 in response to FLG22 through ethylene signaling. This leads to reduced activation of AtERF104 and increased susceptibility to bacterial and fungal pathogen (Bethke et al., 2009). Hence, the phosphorylation is essential for activation of AP2/ERFs for different purposes, but limited information is available on the entire phosphorylation cascade. Both Up and downstream components of MAPKs; other interacting, inducing and inhibiting partners of MAPKs are unknown for most of the AP2/ERFs. Therefore, genetic alteration in plants through overexpression or silencing of MAPKs poses great uncertainty and so proper study is necessary to understand their role in activation of AP2/ERFs.

### N-end rule recognition

**Figure 1:**
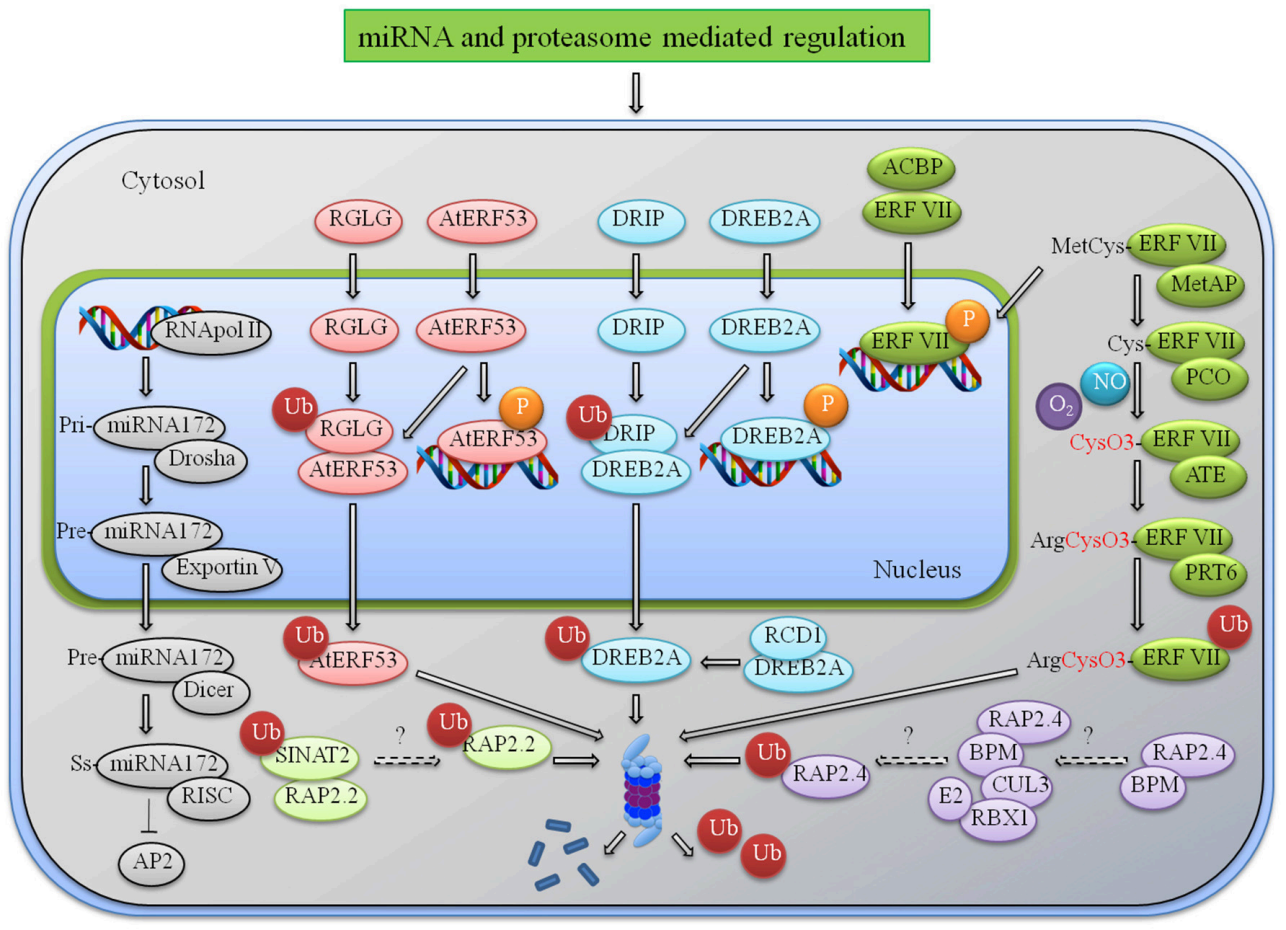
**miRNA and proteasome mediated regulation of AP2/ERFs**. An illustrative model representing the regulation of AP2/ERFs at various levels during multiple stress responses. After onset of stress multiple ERFs are induced and they in turn interact with the cis elements present in the promoter of stress-responsive genes and provide tolerance to various stress. Under normal condition they induce some negative effects like reduced height and senescence that needs to check by indegenous system. Degradation or translational repression by miRNA172 is one example. Other mechanisms include proteasome mediated degradation by different E3 ligases. RGLG interacts with AtERF53 and DRIP interacts with DREB to regulate their expression under normal condition. N-end rule mediated degradation of group VII ERFs under normal (no hypoxia) condition is another mechanism. RAP2.2 and RAP2.4 are also processed for proteasome degradation by SINAT2 and CUL3 ligases. Abbreviations: RGL, RalGDS-like; PRT6, Proteolysis6; SINAT, Seven in absentia 2 of *Arabidopsis thaliana*; DRIP, DREB2A-interacting protein; RCD1, Radical-induced cell death 1; BPM, BTB/POZ-MATH (bric-a-brac/POX virus and zinc finger—meprin and TRAF homology); RBX1, RING-box protein 1; CUL3, Cullin3; Met AP, methionine aminopeptidase; ATE, arginine transferase; PRT6, Proteolysis 6; RISC, RNA-induced silencing complex.

Many group VII hypoxia responsive AP2/ERFs are regulated by this unique N-end rule pathway. Under control condition these AP2/ERFs such as HRE1/2 except RAP2.12 are modified at the N-terminal by Pco1/2 (Plant Cysteine Oxidase 1) which are then recognized and degraded by N recognins ATE1/2 (Arginyl tRNA Protein Transferases) & PRT6 (Proteolysis 6) as shown in Figure 1. The N-end rule pathway is O_2_ and NO dependent in which N-terminal methionine is cleaved by methionine amino-peptidase and Cys which is oxidized by the process that depends upon availability of O_2_. This can be involved in the acidification of cytosol, change in redox state, ROS or NOS mode of action and enzymatic activities (Pucciariello and Perata, 2016). Under oxygen deprivation these TFs become stable and activate hypoxia responsive genes. RAP2.12 escape from N-end degradation by interacting with ankyrin-domain of plasma membrane linked ACBPs (Acyl-CoA binding protein). This interaction retains RAP2.12 at membrane site ensuring a minimum reservoir during the fast proteasomal degradation (Li et al., 2008; Licausi et al., 2011). Similarly ACBP4 is the interacting partner of RAP2.3 in the cytosol and nuclear membrane (Li et al., 2008). Under normal conditions Hypoxia Response Attenuator1 (HRA1) interacts with RAP2.12 to change and repress transcriptionally hypoxia-responsive genes (Giuntoli et al., 2014). N-end rule pathway can be targeted specifically to regulate certain conditions like hypoxia and submergence response. But Submergence-responsive (Sub1) is not regulated by N-end rule though it possesses the same N-terminal recognition sequence (Gibbs et al., 2011). It raises the question whether a single pathway can be applied universally to regulate a particular response due to N-end rule recognition. The further exploration of hypoxia-related group VII ERFs and role of N-end rule recognition will provide better understanding of role of this family of protein under oxygen deprived condition.

### Ubiquitination

Ubiquitination is another post-translational mechanism through which AP2/ERFs are regulated. It may affect proteins in number of ways, either it process them for proteasomal-mediated degradation, or change cellular location, or alter their activity or enhance/prevent interaction of protein. Adding to the list of tight regulation of DREBs is the ubiquitination by DRIP1/2 (DREB2a-Interacting Protein1) under non-stressed condition. These are RING E3 ligases that interact with DREB2A in the nucleus and leads to its ubiquitination in the cytosol (Qin et al., 2008). RING domain containing ligase seems to majorly target AP2/ERFs for protein degradation as seen in Figure 1. For example SINAT2 (SEVEN IN ABSENTIA OF ARABIDOPSIS2) process AtRAP2.2 and RGL1/2 (RalGDS-like) process AtERF53 for proteasome-mediated degradation (Welsch et al., 2007; Cheng et al., 2012). Another cullin-based E3-ligases targets RAP2.4a to 26S proteasome by interacting with the MATH (Meprin and TRAF-tumor necrosis factor receptor-associated factor homology) domain of BTP/POZ (Broad complex, Tram track, bric-a-brac/POX virus and Zinc finger) proteins (Weber and Hellmann, 2009). Regulation of AP2/ERFs through proteasome-mediated degradation to obtain a balance in the stressed and control condition is not very well-understood. Also how protein turnover rate is maintained the under different environmental/developmental condition is a major area of future research.

### Protein–protein interaction

AP2/ERFs physically interact with other proteins, which helps in localization, stability, abundance, transcriptional activity and target specificity. An example for protein-protein interaction includes DREB1A, DREB2A, and DREB2C proteins that specifically interact with the AREB1/ABF2 (ABRE-binding protein/ABRE-binding factor) and AREB2/ABF4 proteins to regulate ABA response (Lee et al., 2010). Huge number of interactions of other proteins with AP2/ ERF has been reported to mediate various responses (Buttner and Singh, 1997; Diaz-Martin et al., 2005; Song et al., 2005; Kagale and Rozwadowski, 2011; Wang X. H. et al., 2014). Some are involved in biotic responses (GmERF5 interact with GmbHLH and eukaryotic translation initiation factor GmEIF to provide *Phytophthora sojae* resistance in soyabean) while some are involved in development (OsERF3 and WOX11 interact with cytokinin-responsive gene RR2 to regulate crown root development in rice) (Dong et al., 2015; Zhao et al., 2015). Intensive complicated interaction is observed in certain AP2/ERFs such as AtERF5 physically interacts with AtERF6, AtERF8, SCL13, MPK3, and MPK6 like proteins to regulate wide array of responses. On one hand it positively regulate salicylic acid signaling and plant defense against the bacterial pathogen *P. Syringae* and fungal pathogen *B. cinerea* while on the other hand it negatively regulate chitin signaling and plant defense against the fungal pathogen *Alternaria brassicicola* (Moffat et al., 2012; Son et al., 2012). Yeast 2 hybrid, Co-immunoprecipitation and Bimolecular fluorescence complementation assays should be performed extensively not only in model plants but in other economically important plants to identify the vast network of AP2/ERF proteins and their interacting partners under different conditions. This is not an easy task but differential binding affinity in closely related species asks the need to perform these assays prior to genetic modifications in plants.

## Domain regulation of AP2/ERFs

### Affect of DNA binding domain

**Figure 2:**
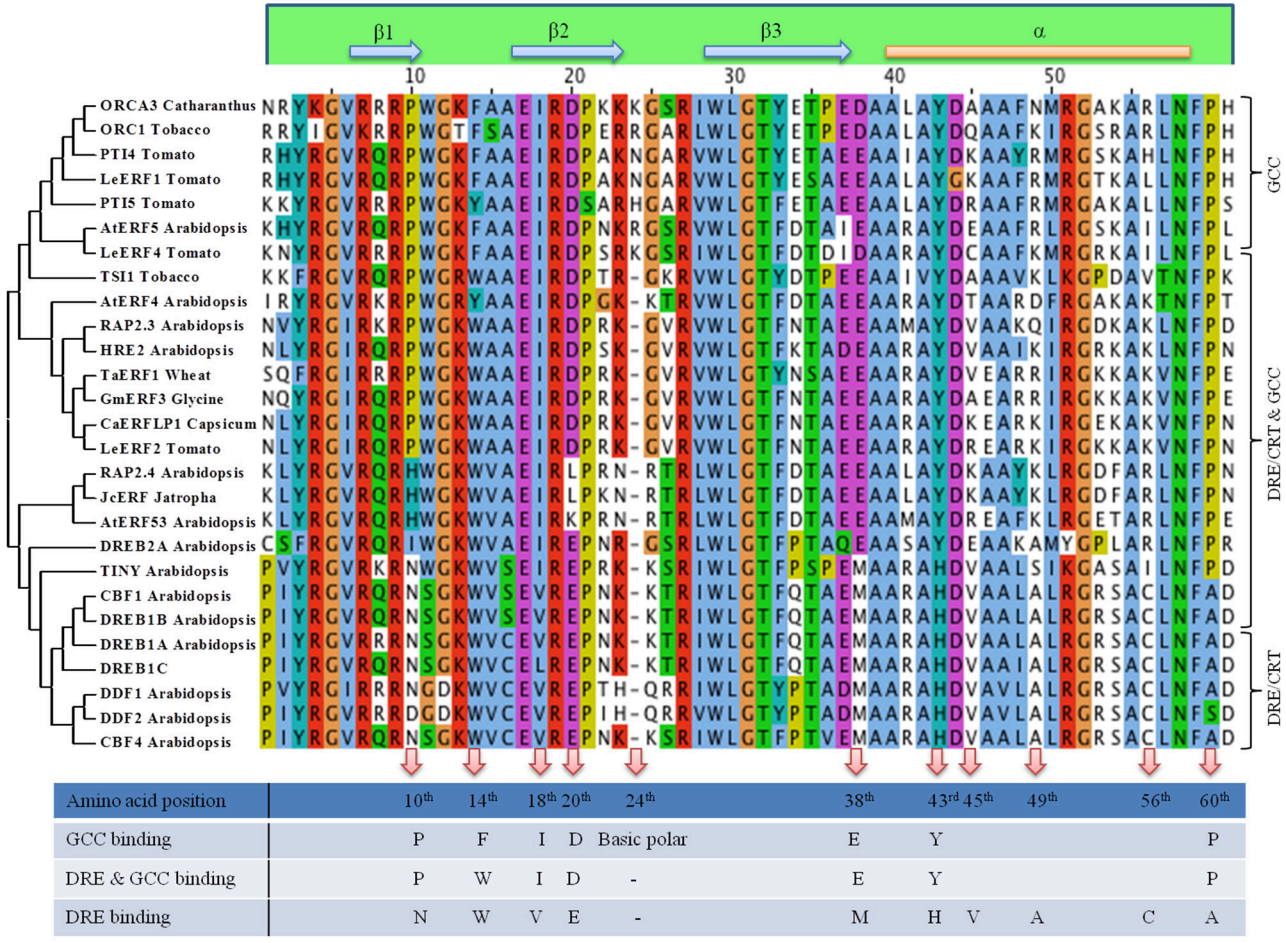
**Clustal and Phylogenetic analysis of DNA binding domain of AP2/ERF**. Based on the conserved amino acid sequences present in the DNA binding domain of AP2/ERFs Clustal Omega multiple alignment was generated. Three groups have been considered, one group that binds to both GCC box cis element, one group that binds to DRE cis element and one group that binds to both DRE and GCC cis element. Phylogenetic clustering was done using MEGA6 neighbor joining algorithm. Amino acids that may regulate specific or multiple binding to downstream cis element is also shown.

Approximately 60 amino acid DBD of AP2/ERFs consists of a three-stranded β-sheet and one α-helix running almost parallel to the β-sheet. The Arg and Trp residues of the β-sheet are necessary for the contact with DNA (Allen et al., 1998). Divergent DNA-binding specificities are seen in many ERFs affecting their affinity to different cis elements. Alignment of the DBD of AP2/ERFs based on their differential binding along with their phylogenetic analysis is shown in Figure 2. With our current knowledge based up on their characterization the AP2/ERFs that bind specifically to GCC motif carries an extra amino acid (basic polar) at 24th position of DBD accounting for 61 amino acid long DBD. While presence of Glu at 20th position and Ala at 49th position of DBD seems to favor DRE binding. Conserved Val (15th position) is also crucial in the regulation of the binding activity of DREB1A to the DRE cis-element (Cao et al., 2001). DRE binding AP2/ERFs consist of 2 different clades with one clade containing DREB2s and rest in other clade. Amino acid alignment of DREB2s showed more similarity with the AP2/ERFs that binds to GCC or GCC and DRE. At 18th, 38th, 43rd, and 60th position DREB2s showed similarity with GCC and GCC and DRE binding ERFs but not with other clade of DRE binding ERFs (Figure 2). Specific amino acid in DBD that regulate binding to both the cis elements is still not known but it is highly possible that motifs present outside DBD may regulate this differential binding. There are reports of modification in DBD which alters binding affinity of certain ERFs. Substitution of a conserved Arg to Lys in the first β-strand of AtERF189 which normally interacts with P-box (CCGCCCTCCA) was able to bind with the second GC pair of the GCC box cis element (AGAGCCGCCA). But an increased number of basic amino acids in the first two β-strands of ORCA3 allow recognition of more than one cis-element like GCC box, P box, and CS1 (TAGACCGCCT) presumably via increased electrostatic interactions with the negatively charged phosphate backbone of DNA (Shoji et al., 2013). In a recent report, it is shown that Arg in 156th position in DBD shows the highest binding affinity to the GCC box (Zhuang et al., 2016). Apart from DNA binding ability, AP2/ERF DBD is reported to be involved in protein-protein interactions. DBD of DRN and DRNL interact with PAS-like domain of HD-ZIP proteins Phavoluta, Phabulosa, Revoluta, and AtHB8. These complexes act as a transcriptional unit in the control of embryo patterning (Chandler et al., 2007). Though AP2 DBD is highly conserved but evolutionary modification is seen in different members of this family ranging from number of DBD (2/1) to linker domains. Other conserved motifs span the entire amino acid sequence of AP2/ERFs (Nakano et al., 2006). Therefore, alteration of DNA binding affinity/specificity of AP2/ERFs through modification of DBD may require attention from many amino acids and motifs present outside of DBD.

### Activation and repression domains

TFs interact with their DBD to the *cis*-motifs presents in the promoter of downstream genes and then activation or repressor domains further enhance or suppress expression of them. As studied earlier DREBs are extensively studied for their complex regulation. Another mechanism, through which DREB2A expression is regulated, is by hydrophilic Ser/Thr-rich negative regulatory domain (NRD) adjacent to DBD that makes DREB2A unstable under normal condition. Deletion of NRD converts DREB2A into its constitutively active form whose overexpression displays reduced growth but improved drought and salt tolerance (Sakuma et al., 2006). Tiwari et al. (2012) identified a potential transactivation domain in AtERF98 as EDLL motif (xxExxxxDxxxLxxxL) having acidic amino acids and hydrophobic leucines. EDLL motif of certain AP2/ERFs like ORA59, AtERF98, AtERF1, and AtERF15 physically interact with ACID domain of MED25 to regulate Jasmonate and stress signaling (Ou et al., 2011; Cevik et al., 2012). In another report, acidic C-terminal region of ERF2, as well as both N-terminal and C-terminal regions of ERF4 from tobacco, act as transactivation domains which regulate activation of downstream genes (Ohta et al., 2000). Apart from the activation domains, certain negative or repressor domains such as C-terminal EAR (L/FDLNL/F(x)P) motif are present in many AP2/ERFs (Ohta et al., 2001; Zeng et al., 2015; Park and Grabau, 2016). Another repression motif BRD (L/VR/KLFGVXM/V/L) is identified in B3 DBD containing TFs like AtRAV1 and AtRAV2 (Ikeda and Ohme-Takagi, 2009). EAR or BRD motif interacts with the transcriptional corepressors TOPLESS (TPL) and TOPLESS-RELATED (TPR) to suppress the transcription of downstream target genes (Causier et al., 2012). The DREB2A/2B/2C contains a RIM (RCD1-interacting motif-FDXXELLXXLN) motif that interacts with RST (RCD1–SRO–TAF4 in which TAF4 stands for TATA-box-binding protein-associated factor 4) domain of RCD1 (Radical- Induced Cell Death 1) to regulate proteasome-mediated degradation under normal conditions. Under heat stress expression of RCD1 is contained, leading to increased expression of DREBs. DREBs without RIM motif like DREB2E and alternative splice variant DREB2A.2 could not interact with RCD1 (Vainonen et al., 2012). Activation or repression of downstream genes through these motifs may positively or negatively influence different responses. Some of the AP2/ERFs (with a conserved C-terminal EAR repressor domain) like GmERF4 (enhanced salt & drought tolerance), GmERF5 (Enhanced *P. sojae* resistance), GmERF6 (enhanced drought tolerance), and NtERF3 (increased resistance to TMV) positively regulates stress response while AtERF7 (reduced sensitivity of guard cells to ABA and increased transpirational water loss) negatively regulates stress response (Fischer and Droge-Laser, 2004; Song et al., 2005; Zhang et al., 2010; Zhai et al., 2013; Dong et al., 2015). Therefore, identification of activation or repressor domain, downstream target genes, domain regulation of AP2/ERFs and their role in a particular response is necessary so that we could attain success in development of genetically modified crops with minimal unwanted effects.

## Retrograde regulation of AP2/ERFs

### Signal from chloroplast

**Figure 3:**
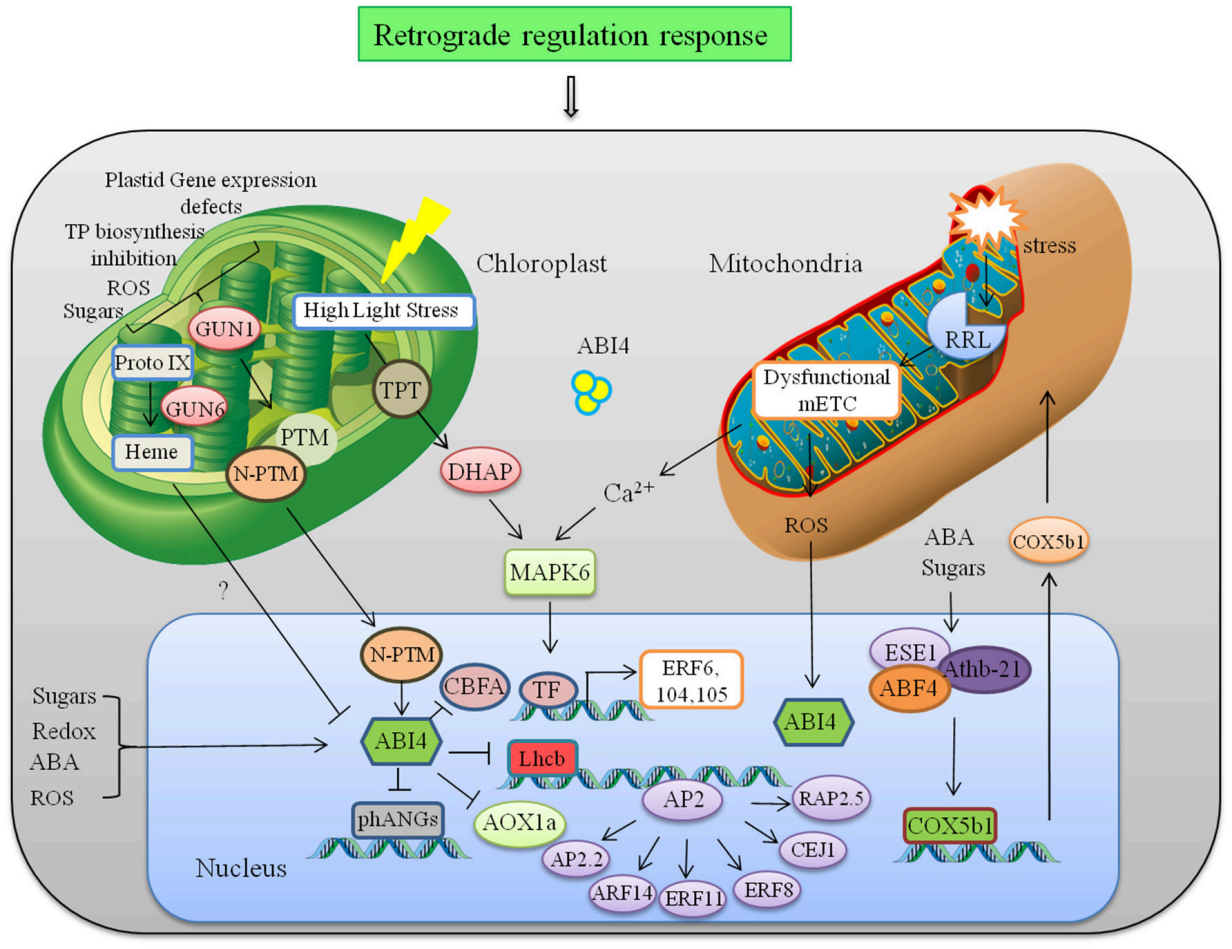
**Retrograde regulation of AP2/ERFs**. Abnormalities in plastid leads to induced expression of GUN1, which mediates retrograde signaling to regulate nuclear gene *ABI4* and photosynthesis related genes like *Lhcb*. ABI4 is also regulated by mitochondrial retrograde signaling through mitochondria located RRL protein. RRL regulate expression of AOX1a to further to maintain ROS homeostasis. Another ERF ESE1 interacts physically with Athb21 and ABF4, all of which interact with the *COX5b1* promoter to regulate respiratory chain complex. Abbreviations: GUN1, genomes uncoupled-1; PTM, Plant homeodomain type transcription factor with transmembrane domain; *phANGs*, Photosynthesis associated nuclear genes; CBFA, (CCAAT binding factor A;), ABI4, ABA-INSENTIVE 4; *Lhcb*, LIGHT HARVESTING COMPLEX B; AOX, Alternative oxidase; ESE1, Ethylene and salt inducible 1; ABF4, ABREbinding factor 4; *COX5b1*, cytochrome c oxidase subunit 5b-1.

Crosstalk between cellular organelles to regulate multiple responses is a quite unexplored field. Data analyzed through graphical Gaussian Models of gene networks and other studies reveal that many members of the AP2/ERF family are involved in integration of signals derived from organelles in retrograde feedback loops and in stress acclimation (Dietz et al., 2010). In this study, *AT2G44940* was identified with the highest score for chloroplast targeting. Schwacke et al. (2007) conducted a bioinformatics search to identify more than 10 nuclear-encoded AP2/ERFs with potential targeting to plastids or mitochondria. Among them *AT2G44940, AT1G77640, AT1G44830, AT1G21910* were showing highest potential to be involved in retrograde signaling. Generally there are 3 plastid to nucleus retrograde signaling pathways, first one includes accumulation of Mg–protoporphyrin IX and downregulation of many genes in *Arabidopsis*, second one represses *Lhcb* expression when plastid gene expression is inhibited and third one involves redox state of the photosynthetic electron transfer chain that affects both photosynthesis-related and stress-related genes (Strand et al., 2003; Nott et al., 2006). In all three pathways a chloroplast-localized PPR protein (pentatricopeptide repeat protein family) GUN1 (genomes uncoupled) and nuclear localized ERF ABI4 (ABA insensitive 4) plays an important role. In this process PTM4 (Plant homeodomain type TF with transmembrane domain) regulates ABI4 by moving out from chloroplast to the nucleus and helping in the transmission of retrograde singals from plastid to ABI4. ABI4 has also been identified as a sugar-insensitive mutant, and sugar signaling through ABI4 has been linked to chloroplast retrograde signaling (Oswald et al., 2001; Finkelstein et al., 2002). Aberrant plastid function in *Arabidopsis* leads to induced expression of GUN1 in plastids, finally leading to ABI4 regulated repression of nuclear-encoded genes (Figure 3). ABI4 binds to the promoter of Lhcb (Light Harvesting Complex B) carrying a CUF1 element (Cab Upstream Factor 1, a G-box element required for retrograde signaling) and inhibits light-induced expression of photosynthetic genes when chloroplast development is arrested (Koussevitzky et al., 2007). ABI4 also regulates CBFA (CCAAT binding factor A), which is a subunit of the Heme activator protein trimeric transcription complex (HAP2/HAP3/HAP5). During various environmental stresses under ABA and ROS accumulation, ABI4 down-regulates CBFA, and allow other TF subunits to enter the transcription complex and improves transcriptional efficiency of stress responsive genes instantaneously (Zhang et al., 2013). Characterization of GUN mutant (gun6) showed that increased accumulation of heme which is a positive regulator of the genes associated with photosynthesis by inhibiting the expression of ABI4. Heme not only have an important role in signaling in other organism but also known to be actively transported out of the plastids (Thomas and Weinstein, 1990; Von Gromoff et al., 2008). Apart from that, transcript of several AP2/ERFs like *AtERF6, AtERF104, RRTF1*, and *AtERF105* were involved in fast retrograde response when low-light acclimated *Arabidopsis* plants were transferred to high light (Khandelwal et al., 2008; Vogel et al., 2014). It is proposed that this transfer accumulate higher levels of DHAP (dihydroxyacetone phosphate) that is transferred from chloroplast to cytosol which activate protein phosphorylation cascades regulated by MPK6. Phosphorylation of constitutive but inactive AP2/ERF initiates downstream HSPs (Heat Shock Proteins) and kinases for acclimation response (Vogel et al., 2014).

### Signals from mitochondria

Mitochondrial signal also regulates many AP2/ERFs through retrograde mechanism (Shaikhali et al., 2008; Mishra et al., 2015). ABI4 is not only involved in chloroplast retrograde signaling but also regulated transcriptionally through mitochondrial retrograde system (Yao et al., 2015). Mitochondrial stress leads to disruption of mitochondria-localized protein RRL (Retarded root growth-like) that causes impaired mitochondrial electron transport chain. It leads to reduced ABA-stimulated ROS production that induces expression of ABI4 in nucleus. *ABI4* regulates expression of alternative oxidase (AOX1a) in the alternative respiratory pathway and is involved in seed germination and seedling growth (Figure 3). Another example of mitochondrial retrograde regulation involves expression of *COX5b-1 (cytochrome c oxidase subunit 5b-1)* gene that encodes an isoform of the cytochrome c oxidase zinc binding subunit involved in respiratory chain complex (Comelli et al., 2012). B-3 subgroup of AP2/ERF ESE1 (Early Somatic Embryogenesis 1) interacts with the distal B-like elements CCACTTG induced in response to ABA while HD-Zip protein Athb-21 interacts with ATCATT elements induced in response to carbohydrates in *COX5b-1* promoter. ESE1 and Athb-21 interacts physically with AREB2/ABF4, which binds to the G-box which is required for expression of the *COX5b-1* gene (Figure 3). Vogel et al. (2012) showed that many AP2/ERF such as ARF14, ERF11, ERF8, CEJ1, RAP2.2, and AP2-2 are likely to be involved in retrograde adjustment of nuclear gene expression. Retrograde and inter-organelle regulation is still an unexplored field and much information is required to fully understand the underlying mechanism. In context of the current knowledge photosynthesis-associated nuclear genes and nuclear respiratory complex genes are key components of this retrograde regulation.The net response of any stress is the cumulative interplay of plastid and mitochondria signaling toward nucleus and vice-versa. Therefore, understanding the role of AP2/ERFs in organelle signaling and their cross talk through retrograde signaling will further help us to manipulate the developmental, metabolic, and environmental need in different plants.

## Regulation of AP2/ERFs by PGR (plant growth regulators)

### Ethylene-mediated regulation

Ethylene signaling is an essential component involved in various development processes and stress responses. AP2/ERFs are highly responsive to these signaling cascades (Muller and Munne-Bosch, 2015). Receptors such as ETR1/2 (Ethylene Response1/2), ERS1/2 (Ethylene Response Sensor1/2), and EIN4 (Ethylene Insensitive4) located on the membrane of endoplasmic reticulum mainly perceive ethylene (Lacey and Binder, 2014). Downstream components of this signaling pathway includes CTR1 (Constitutive Triple Response1), EIN2/3 and ERFs (Kendrick and Chang, 2008; Stepanova and Alonso, 2009). Ethylene signaling regulates various ERFs such as AtERF1 (Cheng et al., 2013), AtERF4 (Yang et al., 2005), CarERF116 (Deokar et al., 2015), LchERF (Wu et al., 2014), and JcERF1 (Yang et al., 2014). CTR1and EIN2 both negatively and positively regulates ethylene signaling. Kinase activity of CTR1 is activated in absence of ethylene which phosphorylates the C-terminal of EIN2 preventing it from entering the nucleus (Kieber et al., 1993; Huang et al., 2003; Gao et al., 2008; Bisson and Groth, 2010; Ju et al., 2012). The absence of ethylene promotes interaction of EIN2 with F-box proteins ETP1/2 (Ethylene Insensitive2-Targeting Protein1/2) leading to reduced EIN2 level (Qiao et al., 2009). Under high ethylene level, CTR1 is inactivated and EIN2 is translocated to the nucleus where it directly or indirectly leads to the activation of EIN3 (Ju et al., 2012). Ethylene response is also enhanced by interaction of EIN2 with ECIP1 (EIN2 C-terminus Interacting Protein 1) an MA3 domain-containing protein (Lei et al., 2011). Ethylene also promotes accumulation of EIN3 that in turn initiates activation or repression of downstream stress responsive genes (An et al., 2010). EIN3 level is reduced in the absence of ethylene by interacting with F-box proteins EBF1/2 (Ethylene Insensitive3-Binding F-Box Protein1). Many waterlogging and submergence responsive genes are regulated by ethylene (Phukan et al., 2014, 2015; van Veen et al., 2014). EIN3 is reported to directly interact with SK1/2 (Snorkel1/2) that helps escape flooding by rapid ethylene and GA-mediated internode elongation (Hattori et al., 2009). AtRAP2.2 promotes activation of genes involved in ethylene biosynthesis such as ACS7 and ACO1 (Hinz et al., 2010). The *Sub1A (Submergence 1)* and *Sub1C* from rice are induced by ethylene but acts antagonistically to each other in response to flooding (Pena-Castro et al., 2011). Therefore, ethylene is an important upstream and downstream component of AP2/ERF mediated plant responsive pathways.

### ABA-mediated regulation

Many AP2/ERFs are responsive to ABA signaling. Apart from ABI4 that is involved in retrograde regulation as stated above, ABA-responsive AP2/ERFs regulate various responses. AtERF1/6 is negatively regulated by ABA (Cheng et al., 2013; Sewelam et al., 2013) while JERF1/3 (Wu et al., 2007, 2008), TaERF1 (Xu et al., 2007), GmERF3 (Zhang et al., 2009), CsERF (Ma et al., 2014), LchERF (Wu et al., 2014), and CarERF116 (Deokar et al., 2015) are positively regulated by ABA. It is reported that waterlogging leads to induced level of ABA that activates ERF RAP2.6 that provides enhanced oxidative stress tolerance in *Arabidopsis* (Liu et al., 2012). Importance of ABA-mediated regulation of ERF is highlighted from the fact that overexpression of AtERF4/7 inhibits ABA response and provide stress tolerance (Song et al., 2005; Yang et al., 2005). Although induction of AtERF1 is negatively regulated by ABA but its overexpression leads to increased ABA accumulation and proline which might be the one of the reason for enhanced drought, salt, and heat stress tolerance in transgenic lines (Cheng et al., 2013). JERF1 when overexpressed in tobacco activates ABA biosynthesis related genes such as *NtSDR (short-chain dehydrogenase/reductase)* and provide enhanced cold and salt stress tolerance (Wu et al., 2007). The overexpression of TSRF1 in tobacco increases the expression of *NtSDR* and ABA accumulation (Quan et al., 2010).

### Other hormones

Various PGRs such as GA, SA, JA (Jasmonic acid) BR (brassinosteroid) also affect ERF mediated regulation. Hypoxia responsive HRE1 and HRE2 show ethylene response, but unlike RAP2.2 they lead to inhibition of ethylene signaling and provide GA response (Licausi et al., 2010). On the other hand SUB1A promotes activation of *DWF1/4 (Dwarf1/4)* involved in BR biosynthesis. *DWF1/4* leads to accumulation of DELLA protein such as SLR1 (Slender Rice 1) and SLRL1 (SLR1 like) which inhibits GA responsiveness (Schmitz et al., 2013). AtERF6 is involved in complex crosstalk of ethylene and gibberellin/DELLA pathway. Under osmotic stress, AtERF6 activates *Gibbereln2-Oxidase6* that leads to accumulation of DELLA protein which in turn suppresses GA responses. It was also found that AtERF6 induction was independent of EIN3 (Dubois et al., 2013). GA3 positively regulates the expression of MsERF8 and CaERF116 (Chen et al., 2012). It is reported that JA-mediated leaf senescence is promoted by ethylene. *SUB1A* delays the process of leaf senescence by inhibiting ethylene, JA and SA during stress. JA positively regulates expression of many ERFs such as Tsi1 (Park et al., 2001), OPBP1 (Guo and Ecker, 2004), JERF1 (Wu et al., 2007, 2008), AtERF1 (Cheng et al., 2013), GmERF3 (Zhang et al., 2009), AtERF6 (Sewelam et al., 2013), LcERF054 (Sun et al., 2014), and PsAP2 (Mishra et al., 2015). AtERF1 is considered to a key component in the ethylene/JA mediated defense response in *Arabidopsis* (Lorenzo et al., 2002). Activation of *AtERF1*under different stress conditions such as drought, salt, and heat which requires both ethylene and JA. JA-insensitive mutant failed to activate *AtERF1* expression under stress conditions (Cheng et al., 2013). SA is an important plant defense hormone which positively regulates many ERFs such as TSRF1 (Huang et al., 2004), TaERF1 (Xu et al., 2007), GmERF3 (Zhang et al., 2009), MsERF8 (Chen et al., 2012), AtERF6 (Sewelam et al., 2013), and CarERF116 (Deokar et al., 2015) while it negatively regulates SodERF3 (Trujillo et al., 2008) and CsERF (Ma et al., 2014). JA cross-talk with SA to fine tune plant immune signaling network. It was reported that SA antagonizes certain JA-responsive genes, partly by *ORA59* mediated transcriptional activation. The ERFs regulate the expression of ethylene and JA-responsive genes by targeting GCC box present in their promoters. It seems that GCC-box is sufficient for SA-mediated suppression of JA-responsive gene expression (Caarls et al., 2016).

## Crop improvement by regulating multiple responses through AP2/ERFs

Crop yield is severely affected because of various biotic and abiotic environmental factors. Over the last decade, AP2/ERF proteins have become the subject of intensive research activity in crops due to their involvement in a variety of biological processes. There are many reports in which significant results have been obtained through manipulation and regulation of AP2/ERF-like TFs. In the current scenario even commercialization of transgenics could be possible, but as stated above proper scrutiny at the every level is entirely necessary. As AP2/ERFs can regulate multiple responses in a simultaneous and cooperative manner they can be targeted for improved variety development which may deliver multiple tolerance traits to a single breed. Some of the recent findings are mentioned below.

### Rice

It is one of the worldr's most important staple food crops. Generally half of the total rice cultivation is affected by environmental stress decreasing crop yield and quality. Also, it is a model plant where significant research has been done, which can be explored for generation of improved breeds with very limited unwanted traits. In a study using phylogenetic analysis of the rice genome, 170 AP2/ERF family genes were identified and divided into 11 groups, including four broad groups (AP2, ERF, DREB, and RAV), 10 subgroups, and two soloists (Rashid et al., 2012). *SUB1A* gene encoding an AP2 domain TF restricts GA functioning during sustained submergence in rice. *SUB1A* gene enhances accumulation of GA repressors SLR1 and SLRL1, thus limiting underwater internode elongation and augmenting submergence survival (Fukao and Bailey-Serres, 2008). Snorkel1 and Snorkel2 (SK1 and SK2) were identified to have an important role in internode elongation mainly post-mitotic expansion of differentiating cells separated from the region of intercalary meristem around node. SK1 and SK2 were suggested to trigger internode elongation via GA in response to rising water level (Hattori et al., 2009). By contrast, OsEATB was found to restrain GA responsiveness during the internode elongation process by down-regulating the expression of the GA biosynthetic gene *OsCPS2* (Qi et al., 2011). Overexpression of OsAP37 showed enhanced tolerance to drought in rice plants under vegetative stage and improved grain yield (Oh et al., 2009). OsEREBP1overexpression activates the jasmonate and abscisic acid signaling pathways thus provide enhanced survival to rice plants under abiotic or biotic stress conditions (Jisha et al., 2015). *Multi-Floret Spikelet1 (MFS1)* gene was found to play an important role in regulating determinacy of spikelet meristem and floral organ identity. *MFS1* is associated with unidentified function clade in the family of AP2/ERF which certainly regulates the expression pattern of *Long Sterile Lemma (LSL)* and the *Indeterminate Spikelet1 (IDS1)* like genes *Supernumerary Bract* and *OsIDS1* (Ren et al., 2013). The auxin responsive CROWN ROOTLESS-5 (CRL5) has a function in crown root initiation in rice plants by inducing OsRR1 which act as type-A response regulator in cytokinin signaling. It was known that cytokinin has negative effects on *de novo* auxin-induced root formation (Kitomi et al., 2011). OsERF109 when overexpressed in rice negatively regulates ethylene biosynthesis and drought tolerance (Yu et al., 2017). OsERF71 overexpression in rice leads to root structure modifications, larger aerenchyma and radial root growth which provides improved drought tolerance of shoots. OsERF71 activate various stress responsive, cell wall-related and lignin biosynthesis associated genes causing root structural changes in rice (Lee et al., 2016). In a recent report OsERF115 target GCC box of *OsNF-YB1* promoter to regulate endosperm development and grain filling in rice. *OsNF-YB1* is specific to the aleurone layer of developing endosperms (Xu et al., 2016). CRISPR/Cas9-Targeted Mutagenesis has been used to generate rice lines with enhanced blast resistance through engineering OsERF922 mutation (Wang F. et al., 2016).

### Wheat

As rice, wheat is also important crop and cultivated all over the world. Several ERFs have been characterized from wheat whose regulation could potentially be used for improved variety development. TaPIE1 when over-expressed in wheat displayed significantly increased resistance to fungal pathogen *Rhizoctonia cerealis* and freezing stresses. TaPIE1 (Pathogen-Induced ERF1) transgenic wheat showed reduced electrolyte leakage and H_2_O_2_ content, while increased soluble sugar and proline content (Zhu et al., 2014). Another ERF, TaERF3 led to increased tolerance to salt and drought stresses. In TaERF3 overexpressing transgenic lines increased accumulation of proline was observed. These lines also inhibited chlorophyll degradation, H_2_O_2_ formation and stomatal conductance under both stresses. TaERF3 could interact specifically with the GCC-box *cis*-element present in the promoters of seven stress-responsive genes namely *BG3 (*β*-glucans 3), Chit1 (Chitinase 1), RAB18 (ras-related protein 18), LEA3 (Late embryogenesis abundant 3), TIP2 (Delta tonoplast intrinsic protein 2), POX2 (Peroxidase 2)*, and *GST6 (Glutathione S-Transferase 6)* while could not bind with DRE *cis*-element (Rong et al., 2014). Overexpression of TaPIEP1 in wheat, which is induced in response to pathogen, confers increased resistance to fungal pathogen *Bipolaris sorokiniana*. The transgenic lines showed increased expression levels of certain defense-related genes in the ET/JA pathways, which might be responsible for enhanced disease resistance (Dong et al., 2010).

### Tomato

Tomatoes have been modified for slowing down the ripening process using genetic engineering like *Flavr Savr*, though none are commercially available now. But there are many recent developments made in this area till then. In transgenic tomato JREs, when overexpressed and silenced significantly affected Steroidal glycoalkaloids accumulation and expression of genes involved in its biosynthesis including the upstream mevalonate pathway. One of them, JRE4 binds with two structurally related elements, GCC box-like P box and the GCC box, which are also found on the promoters of biosynthetic genes like sterol reductase and glycoalkaloid metabolism 5 (Thagun et al., 2016). Another TF from tomato SlERF52 functions in flower pedicel abscission. Before the abscission stimulus, SlERF52 regulates the expression of pedicel abscission zone specific TFs, like tomato Wuschel Homolog, Goblet and Lateral Suppressor, which may lead to regulation of meristematic activities in pedicel abscission zones (Nakano et al., 2014). The tomato ERF Pti5 (Pto-interacting protein 5) contributes to potato aphid resistance in tomato as VIGS (Virus Induced Gene Silencing) of Pti5 enhanced aphid population growth (Wu et al., 2015). An extensive study of ERFs on ripening-impaired tomato mutants showed potential role of ERFs in Fruit Ripening in Tomato (Liu et al., 2016). When SlERF5 was overexpressed in tomato, plants showed increased tolerance to drought and salt stress and enhanced levels of relative water content (Pan et al., 2012). SlERF5 overexpressed tomato plants also accumulated higher levels of defense gene *PR5 (Pathogenesis related 5)* and displayed better tolerance to bacterial pathogen *Ralstonia solanacearum*. Constitutive expression of *SlRAV2* in tomato also led to enhanced tolerance to *R. solanacearum* bacterial wilt and increased expression of *SlERF5* and *PR5* genes (Li et al., 2011). TERF2/LeERF2 when overexpressed in tomato, controls ethylene production and showed enhanced freezing tolerance (Zhang and Huang, 2010). Many ERFs from different cultivars of tomato have been identified which may be responsible for the defense mechanisms in response to Tomato yellow leaf curly virus (Huang Y. et al., 2016).

### Arabidopsis

Lots of research and literature surrounding AP2/ERF are available for this model plant. Therefore, we are highlighting only some recent advances made in this area. AP2/ERFs positively/negatively regulate various processes to maintain proper homeostasis during stress and favorable conditions. AtERF019 overexpression delays flowering, plant growth and senescence in *Arabidopsis* thus impart drought tolerance. Genes involved in stress response such as *BCAT3 (branched-chain-amino-acid aminotransferase3)* and the zinc finger *oxidative stress 2* are predicted targets of this ERF (Scarpeci et al., 2016). Recently reported AtERF105 provides cold tolerance by mediating CBF regulon in *Arabidopsis* (Bolt et al., 2017). AtERF014 is induced by both *Pseudomonas syringae* pv. tomato and *Botrytis cinerea* but it positively regulates *P. syringae* resistance while negatively regulates *B. cinerea* resistance in *Arabidopsis*. Overexpression of AtERF014 induces activation of SA-responsive *AtPR1/5* genes while suppresses JA-ethylene responsive *AtPDF1.2* (Zhang et al., 2016). Similarly AtERF15 is induced by *P. syringae, B. cinerea*, SA and JA but it provided enhanced resistance to both the pathogens in *Arabidopsis* (Zhang et al., 2015). Several JA/ethylene responsive ERFs also provide plant immunity in *Arabidopsis* such as AtERF1, AtERF6, ORA59, and AtERF96 (Huang P. Y. et al., 2016). AtERF96 provides increased resistance to *B. cinerea* and *Pectobacterium carotovorum* in *Arabidopsis*. It specifically interacts with the GCC box present in the promoters of defense responsive genes such as *PDF1.2a, PR3/4*, and *ORA59* to modulate those responses (Catinot et al., 2016). AtERF96 has also been shown to be ABA-responsive. Overexpression lines significantly have elevated levels of ABA-responsive genes such as *ABI5, RD29A, ABF4, ABF3, P5CS*, and *COR15A*. AtERF96 overexpressed lines showed hypersensitivity to ABA and had reduced stomatal aperture (Wang X. et al., 2016). Group VII ERFs are responsive to hypoxia and are regulated through N-end rule pathway such as RAP2.2 and RAP2.12 (Gasch et al., 2016). But a recent report showed that under normoxia oxygen-dependent AtRAP2.12 stability plays a key role in central metabolic processes to maintain growth and development in *Arabidopsis* (Paul M. V. et al., 2016). Stem epidermis-specific WRI4 (Wrinkled4) regulates biosynthesis of cuticular wax in *Arabidopsis* stems by interacting promoters of various wax-biosynthesis related genes such as *(LACS) 1long-chain acyl-CoA synthetase1, PAS2 (Pasticcino2), KCR1 (*β*-ketoacyl CoA reductase1), ECR (trans-2,3-enoyl-CoA reductase)*, and *WSD1 (bifunctional wax synthase/acyl-CoA: Diacylglycerol acyltransferase)*. Wax layer in turn protects plants from high irradiance, desiccation and UV radiation (Park et al., 2016). AtERF11 leads to internode elongation by positively regulating both GA biosynthesis and GA signaling in *Arabidopsis*. Overexpression lines showed induced expression of *GA3ox1* and *GA20ox* genes leading to elevated levels of GA while reduced ethylene levels (Zhou et al., 2016). AtERF11 also acts antagonistically to AtERF6 to regulate mannitol-induced growth inhibition in *Arabidopsis*. AtERF6 actually activates AtERF11, which in turn suppresses AtERF6 activated genes by directly competing for the promoters of downstream genes (Dubois et al., 2015).

### Others

Homologous gene expression is a difficult task as transformation and regeneration protocols differ from one species to other. Few examples of AP2/ERF have been successfully overexpressed in homologous system and found some promising results. GmERF5 is the first soybean EAR motif containing ERF proved to be involved against pathogen infection. GmERF5 overexpressing transgenic soybean enhanced resistance to *P. sojae* and positively regulates the expression of the *pathogenesis-related (PR10, PR1-1*, and *PR10-1)* genes (Dong et al., 2015). EjAP2-1 (AP2/ERF) from *Eriobotrya japonica* is a novel regulator of fruit lignification induced by chilling injury, via interaction with EjMYB (Zeng et al., 2015). Shi et al. (2014) characterized two tomato AP2/ERF genes, SlCRF1 and SlCRF2 (cytokinin response factor) under stress and found that SlCRF1 and SlCRF2 expressed prominently in vascular tissue during development and in response to cytokinin and specific stresses, indicating their importance in plant growth and environmental responses.

## Conclusions

Environmental fluctuation mediated crop yield loss is a major problem around the world. Simultaneous impact of several other stresses increases the susceptibility of plants to many folds. The increasing population size of world also raises the continuous requirement of food crops to sustain their requirement. To overcome these adverse conditions without compromising the growth or yield new insight is required in the field of transgenic and improved variety development. In this review we sum up the current understanding and knowledge of regulations of AP2/ERFs under different conditions. These AP2/ERFs are tightly regulated at different transcriptional and translational level to attain a homeostasis during non-stressed conditions. AP2/ERFs are in prime focus in context of generating improved varities, as they are promising candidates for studying diverse network involved in development, metabolic and stress responses in plants. Because of their ability to bind with multiple *cis*-elements, allows them to regulate these diverse set of responses simultaneously. One important fact is that with so many positive effects, they also negatively influence many processes which are indeed and important for normal metabolism and growth of plants. So regulation of AP2/ERFs and downstream signaling has to be kept under proper check by different mechanisms for fruitful response. We believe that these crosstalks should be studied in detail and future research on AP2/ERFs and their integrated regulatory network should take following points into account:

1. MicroRNA and small RNA-mediated regulation are rarely discussed. MiR172 and MiR156 are involved in AP2 regulated intricate floral organogenesis. Recently legume nodulation during the rhizobia nitrogen-fixation symbiosis has been shown to be regulated by MiR172-AP2 complex. In order to identify additional transcriptional regulators associated with, genome-wide identification of miRNAs and their potential targets should be carried out. It seems highly probable that these do not play alone and other regulators need to be studied for the interpretation of the entire network to understand what regulates these regulators?
2. Many stress-responsive AP2/ERFs would impart growth abnormalities under normal condition if otherwise not regulated properly at post-transcriptional (capping, splicing, and histone modifications) and post-translational level (phosphorylation, ubiquitination, SUMOylation). Therefore, these aspects need to be exploited at a large level so that specific targets in the complex mechanism could be identified, which could be modulated for development of improved varieties.
3. AP2/ERFs activate or repress expression of a particular gene by interacting specifically to the *cis*-elements present in the promoter of that gene. We believe that this specificity to identify a single or multiple *cis*-elements is regulated not only by the rare anomalies in the DBD but also by the motifs flanking it. These bindings are also influenced by protein-protein interactions through different domains. So these domains and motifs, which lead to specificity or plasticity in the interaction of AP2/ERFs with their downstream targets to achieve multiple responses, should be thoroughly studied.
4. Also with recent findings, it is evident that retrograde signaling affects several AP2/ERF-mediated responses. Signaling from mitochondria or chloroplast to the nucleus and its feedback is not properly understood. Therefore, new insight is required in the field of retrograde regulation of AP2/ERFs.
5. Recent molecular tools like CRISPR (Clustered Regularly Interspaced Short Palindromic Repeats) and CRISPR-associated 9 (Cas9) genes could be applied to different non-model plants where functional mutants are not available. CRISPR/cas9 genome editing tool would have an edge over conventional tedious silencing approach (through pART vectors) or VIGS (through TRV vectors) in the coming era. Therefore, this technique could be exploited for functional characterization of *AP2/ERF*s from different plant species.

## Author contributions

UP and GJ has compiled and written the review. VT and RS has edited and finalized the draft.

### Conflict of interest statement

The authors declare that the research was conducted in the absence of any commercial or financial relationships that could be construed as a potential conflict of interest.
